# Supplementary material for: An energizing role for motivation in information-seeking during the early phase of the COVID-19 pandemic
Source: Nat Commun. 2022 Apr 28;13:2310. doi: 10.1038/s41467-022-30011-5 (PMC9050882; doi:10.1038/s41467-022-30011-5)
Supplement: Supplementary file 1 — Supplementary Information [file 41467_2022_30011_MOESM1_ESM.pdf]

Supplementary Information for

An Energizing Role for Motivation in Information-Seeking During the Early Phase of the COVID-19 Pandemic

Yaniv Abir[1*], Caroline B. Marvin[1], Camilla van Geen[2,3], Maya Leshkowitz[4], Ran R. Hassin[5†], Daphna Shohamy[1,2,6†]

[1]Department of Psychology, Columbia University, New York, NY, United States.

[2]Zuckerman Mind Brain Behavior Institute, Columbia University, New York, NY, United States.

[3]Department of Psychology, University of Pennsylvania, Philadelphia, PA, United States.

[4]Department of Cognitive Sciences, The Hebrew University of Jerusalem, Jerusalem, Israel.

[5]Department of Psychology and The Federmann Center for the Study of Rationality, The Hebrew University of Jerusalem, Jerusalem, Israel.

[6]Kavli Institute for Brain Science, Columbia University, New York, NY, United States.

[†]These authors contributed equally to this work.

Author Note

Correspondence concerning this article should be addressed to Yaniv Abir, Columbia University, 3227 Broadway, New York, NY 10027, United States. Email: yaniv.abir@columbia.edu

Supplementary Methods

Data Collection

The study was approved by the Columbia University Institutional Review Board (protocol number AAAS0166). Data was collected twice weekly, on Mondays and Thursdays, between March 11th and May 7th 2020. Participants were recruited through Amazon Mechanical Turk (MTurk) and had to have at least 95% prior task approval ratings. Participant recruitment was managed through cloudresearch.com. Throughout weeks 2-5 of data collection, 400 new participants were recruited on each day of data collection. In the last 3 weeks of collection this number was reduced to 300 per day for budgetary reasons. During the first week of data collection, we were setting up data collection infrastructure, and so participant numbers were lower (652). Altogether, 6135 participants completed session 1. A week after their participation in the first session, participants received an email inviting them to participate in session 2 of the study, which was available on days 7 and 8 after the original session. Overall, 71.48% of eligible participants returned for session 2.

All participants gave informed consent to their participation in this two-session study and were paid $2.30 plus a $2 bonus for successful attentive completion of session 1. Participants were paid $1, plus a guaranteed $2 bonus and a $1 bonus contingent on successful attentive completion of session 2.

Stimuli

A set of short questions and answers was used as stimuli in this experiment. Questions were of three types. 52 *COVID-19-related questions* were sourced from materials published by the World Health Organization, US Centers for Disease Control and Prevention, or the New York Times. Half of these were deemed useful by the authors of the study and half non-useful. 52 *general questions* comprised the second type of questions - half of these were trivia questions drawn from previous studies[1], and half were useful household tips sourced from lists of tips on the internet. Results for these two question types are reported in the main text. Additionally, a third category of questions was included for exploratory purposes. This category comprised 25 *crowdsourced* questions, collected previously by eliciting interesting questions from MTurk participants. 25 additional trivia questions were added to this category as well. Data relating to these crowdsourced questions are not analyzed further in this paper.

Session 1 Procedures and Tasks

The broader study was designed to investigate the effects of individual differences in personality, motivation and context on curiosity and curiosity-driven behavior. Participants first completed three blocks of the waiting task, then completed a question rating task on a subset of 5 questions randomly held out from each question type for each participant. Participants then chose to read between 2 and 20 additional COVID-19-related or general facts. Participants proceeded to fill out a battery of questionnaires. First, affective and mood questions sourced from the STAI questionnaire and Gallup World Survey wellbeing section. Then, participants completed the five-dimensional trait curiosity questionnaire[2], a trait resilience questionnaire[3], a questionnaire assessing affective concerns regarding COVID-19 - the COVID-19 concern measure, and general demographic information, including age, gender, income, command of the English language and geographical location. The fact choice task, resilience questionnaire and some of the questions in the COVID-19 concern measure were added after 3 weeks of data collection. The entirety of data available was used for analysis of each task.

Waiting Task

On each trial of the waiting task, participants were presented with a question. Using their computer mouse, participants had to click one of three buttons - 'Know' if they knew the answer to the question, 'Wait Xs' if they were willing to wait for the answer (with the specific duration for the trial replacing 'X'), or 'Skip' if they were not. An equal number of trials was assigned to each wait duration from the set of 4, 8, 12, 16s, in random order. Participants could respond starting 1500ms after trial onset, to discourage arbitrary mouse presses, and if no response was given within 10s the trial terminated with a message prompting to respond more quickly. If participants chose to wait, an ellipsis was displayed for the specified duration, after which the answer to the question was displayed. Once participants pressed the 'Continue' button (with a minimum answer reading time of 1500ms, maximum 7s), a satisfaction scale appeared on screen. Participants rated their satisfaction with the answer on a scale of 1-5. Participants had 3500ms to register their satisfaction rating. If participants failed to respond to the answer presentation or rate their satisfaction within the specified time window, a message prompting them to respond faster was displayed. If participants reported knowing the answer or chose to skip it the trial was terminated. The inter-trial interval for the task was drawn from a uniform distribution on the 500-1200ms range. See Supplementary Figure 1a for screenshots of the waiting task.

Participants completed three blocks of the waiting task - a COVID-19-related block, a general block, and a crowdsourced block. The first two were counterbalanced in order, the

crowdsourced block was always last. Each block was 2.5 minutes long, regardless of participants' choices. This was explained in the instructions for the task, and participants were encouraged to base their decisions on how interested they were in learning the answer.

Question Rating Task

We chose to measure ratings for a non-overlapping set of questions for each participant to avoid biasing the ratings by previous waiting-task responses and avoid any demand characteristics resulting from being asked to respond twice to the same question.

Participants were presented with the held-out questions from the three question types, one question at a time. Under each question a rating prompt appeared: “The answer to the question above is…”, and below 1-7 Likert scales appeared for the items: ‘...something that would be useful for me to know’, ‘...something that would be useful for others to know’, which constitute our usefulness measures, along with ‘…something that well-informed people would agree on’, ‘...knowable in principle, given enough information’, ‘..something that has an element of randomness’ and ‘...determined by chance factor’. The last four items constitute an epistemicness scale[4] included for the exploratory purpose of assessing the spread of epistemicness ratings in a question set similar to those prevalent in the literature. Results from this scale are not analyzed in this paper.

Session 2 Procedures and Tasks

Participants were eligible to participate in session 2 if their session 1 data were not excluded according to the criteria detailed below, and if they had chosen to wait for at least a single question in the waiting task.

In session two participants first completed the answer recall task, and then were asked to recollect their answers from session 1 to the COVID-19 concern questionnaire. Finally, they repeated all affective questionnaires and the COVID-19 questionnaire regarding their current feelings. The repeated questionnaires were included for exploratory purposes. Participant’s responses were highly correlated with their responses on session 1 and are not further analyzed here.

Answer Recall Task

On each trial of the answer recall task participants were presented with one question whose answer they had waited for the previous week. Questions were presented in random order. Participants first indicated whether they remembered the answer they had read for the

presented question. If they indicated they remember the answer, they were asked to input it into a text field. See Supplementary Figure 1b for screenshots of the answer recall task.

Statistical Analysis

Scoring Memory Recall

Participants' recollected answers were compared to the original answer they had seen. Exact matches (bar capitalization or one letter typing errors) were scored as correct by an R program. A research assistant blind to the research hypotheses and conditions scored the remaining responses as either incorrect, not recalled (e.g. "I don't remember"), partially correct, or correct. 311 responses (0.90%) were flagged as not compatible with instructions. Both partially correct and correct answers are considered as successful recollection in all subsequent analysis, while incorrect and not recalled are considered as recollection failures.

Validating the COVID-19 Concern and Non-Specific Anxiety Measures

We used COVID-19 concern as our main measure of the specific motivational state elicited by COVID-19, and non-specific anxiety as a control measure, accounting for any general negative affect or anxiousness that might explain the link between COVID-19 concern and epistemic behavior. Non-specific anxiety also serves as a control for any general directional transfer that might have occurred between the waiting task and the questionnaires. Since the items comprising the non-specific anxiety measure were completed before the COVID-19 concern items, any anxiety induced by the COVID-19-releated answers in the waiting task should presumably affect the non-specific anxiety measure as it does the COVID-19 concern measure. See the supplementary replication experiment below for a version of the task in which directional transfer was even less likely to occur.

When evaluating the results of the BPCA confirmatory analysis (see main text Method), two variables had very similar loadings on COVID-19 concern and non-specific anxiety, but since they were about COVID-19 they were assigned to the COVID-19 group. Results are similar without these items. We used the unweighted means of each variable group to avoid overfitting. The relation between information-seeking and COVID-19 concern or non-specific anxiety is very similar when using a naive grouping of items, according to the original questionnaire they came from.

We further validated COVID-19 concern and non-specific anxiety measures by relating them to real-life events that we predicted should result in meaningful differences in motivational state. We compared ratings on both measures for participants who experienced job loss, a reduction

in income or self-isolation, with ratings for participants who did not experience these events. Independent samples t-tests with the Welch modification for unequal variances were used to assess the statistical significance of these differences. We also tested the dependence of the two anxiety variables on a measure of social distancing in each participant's state on the day of data collection, derived from mobile phone data collected and analyzed by Unacast[5]. We used a generalized additive model to account for potential non-linearities and allowed intercepts to vary by state. The regression equation can be described in R syntax as following:

COVID-19 concern / non-specific anxiety ~ s(social distancing) + (1 | state)

Supplementary Equation 1

A similar model was fit to assess the change in the COVID-19 concern and non-specific anxiety over the course of data collection.

When conducting dimensionality reduction analyses such as BPCA, it is never straightforward to name the resultant components, as the analysis method is unsupervised and meaning-agnostic. Hence, it is important to validate the resulting measures, as we do above. One should also be clear about the reasoning behind the naming scheme. *COVID-19 concern* was chosen as a term encompassing the affective and motivational relevance of COVID-19 to an individual. *Non-specific anxiety* was chosen to underline the use of this measure as a control for the influence of any non-specific anxiety that may be captured by the COVID-19 concern measure. We do not claim that it is specific to anxiety, indeed it could have plausibly been named *non-specific negative affect* or *mood*. We chose anxiety as we found it more succinct.

Usefulness as Predictor in Regression Models

Usefulness was judged on an ordinal Likert scale with only two ratings made by each participant for each question. Thus, we first fit usefulness ratings with a two-parameter ordinal item response theory (IRT) model to extract judged usefulness estimates on a metric scale, rather than apply an averaging operation to the raw ordinal data[6,7]. The model included a term for the average usefulness of each question, and the average rating of each participant. Using R syntax, this is the IRT model fit to usefulness judgments:

useful_me, useful_others ~ (1 | participant) + (1 | question),

family = ordered(link = “logit”)   Supplementary Equation 2

We chose an ordered-logistic likelihood function for the usefulness ratings, with a separate set of threshold parameters [6] for each of the two usefulness items (useful for me / useful for others),

to allow for different use of the Likert scale for these two items. The model was fit to usefulness ratings using maximum a posteriori (MAP) estimation with the Stan language[8]. We used the MAP estimates for each question's usefulness in all subsequent models in which usefulness is a predictor. These estimates are highly correlated with the raw averages of the ordinal ratings $r=0.99$, $p< 0.001$.

Assessing the incentive value of questions

In addition to the analyses reported in the main text, a simpler validation of our measures and theoretical framework would confirm that people seek information in a cost-benefit-rational manner. The two main predictions would be that information-seeking increases for questions perceived as more useful and decreases with longer wait durations. Focusing on attributes of each question that hold across differences in motivation is commensurate with the lion-share of literature on curiosity, which explores the incentive value of information.

Regression Equations. We fit the data with the following three regression equations to test behavior for cost-benefit rationality. In R syntax they are:

waited ~ wait duration * usefulness * question type +

+ (wait duration * usefulness * question type | participant) + (wait duration | question), family = bernoulli() Supplementary Equation 3

satisfaction ~ wait duration * usefulness * question type +

+ (wait duration * usefulness * question type | participant) + (wait duration | question), family = ordered(link = “logit”) Supplementary Equation 4

recalled ~ wait duration * usefulness * question type +

+ (wait duration * usefulness * question type | participant) + (wait duration | question), family = bernoulli() Supplementary Equation 5

Plotting the Effect of Usefulness and Wait Duration on Waiting. During the waiting task, participants who skip more questions end up completing more trials, as the total length of the task block is fixed. This creates a dependency between participants’ baseline willingness to wait and the number of trials they contribute to the dataset, with over-representation of participants

who tend to skip. When computing the proportion of participants who waited for each question in the stimulus set, participants who tend to skip will contribute more trials to the computed proportions than will participants who tend to wait, thereby lowering the estimated average willingness to wait of the group. When computing marginal proportions for questions from the model described in Supplementary Equation 3, on the other hand, no such problem will arise, since the model adjusts for each participants' baseline willingness to wait. Hence, in Supplementary Figure 2a we used the latter method of computing marginal means for the points representing each question at each duration level. No such problem arises for satisfaction and memory data, as there is no significant correlation between average responses and the number of trials for these measures. Hence, we used simple averages for creating Supplementary Figure 2b,c.

Assessing the Effect of Motivational States on Epistemic Behavior

The following set of three regression equations, given in R syntax, was fit to the data to assess the effects of motivational state, as described in the main text:

waited ~ wait duration + usefulness * question type * COVID-19 concern +

+ usefulness * question type * non-specific anxiety +

+ (wait duration + usefulness * question type | participant) +

+ (wait duration + COVID-19 concern + non-specific anxiety | question),

family = bernoulli() Supplementary Equation 6

satisfaction ~ usefulness * question type * COVID-19 concern + usefulness *

* question type * non-specific anxiety + (usefulness * question type | participant) +

+ (COVID-19 concern + non-specific anxiety | question),

family = ordered(link = “logit”) Supplementary Equation 7

recalled ~ usefulness * question type * COVID-19 concern + usefulness *

* question type * non-specific anxiety + (usefulness * question type | participant) +

+ (COVID-19 concern + non-specific anxiety | question),

family = bernoulli() Supplementary Equation 8

Controlling for List Length in Recollection Data. The tendency to recall an answer may be influenced by the number of answers waited for, which determines the length of the answer list to be remembered. Thus, another control model was fit to recall data, with number of answers waited for as a covariate interacting with question type:

recalled ~ number waited * question type +

+ usefulness * question type * COVID-19 concern +

+ usefulness * question type * non-specific anxiety +

+ (usefulness * question type | participant) +

+ (COVID-19 concern + non-specific anxiety + number waited | question),

family = bernoulli() Supplementary Equation 9

Assessing the Effect of Motivational States on Usefulness Judgments

The regression model fit to usefulness ratings, as described in the main text, can be described by the following R syntax equation:

useful_me, useful_others ~ question type * COVID-19 concern +

+ question type * non-specific anxiety + (1 + question type | participant) +

+ (1+ COVID-19 concern + non-specific anxiety | question),

family = ordered(link = “logit”) Supplementary Equation 10

Here again we fit separate threshold parameters for each usefulness item.

Joint Analysis of Waiting Choices and Usefulness Judgments

The mediation model fit to usefulness judgements and waiting choices, as described in the main text, can be described by the following two regression equations in R syntax:

useful_me, useful_others ~ 1 + question type * COVID-19 concern +

+ (1 + question type | participant) +

+ (1 + COVID-19 concern |question), family = ordered(link = "logit")

Supplementary Equation 11

waited ~ 1 + question type * COVID-19 concern + usefulness +

+ (1 + question type + usefulness | participant) +

+ (1 + COVID-19 concern + usefulness |question), family = bernoulli()

Supplementary Equation 12

Supplementary Equation 11 defines the mediator model and Supplementary Equation 12 defines the outcome model in mediation-analysis parlance. In a regular mediation analysis, raw usefulness ratings would be used as the predictor for the outcome model. However, the experimental design precluded this approach - each individual participant did not rate the usefulness of the same questions presented to them in the waiting task. Hence, predictions from the mediator model were used as the usefulness predictor in the outcome model. The use of predictions instead of ratings both mitigates the incompatibility of the experimental design with mediation analysis, and at the same time accounts for error in the measurement of usefulness in the outcome model (in comparison, in traditional mediation analysis the mediator is treated as error-free in the outcome model).

The joint model was fit using the Stan programming language with the same methods used for the regression models detailed above, but with 3000 samples per chain to allow for sufficient effective posterior sample size, given the more complex model.

Assessing the Effect of Motivational States on Waiting for Answers to Trivia Questions

To determine whether the conclusions of the motivational analyses above generalize to trivia questions, heretofore considered the quintessential example of non-instrumental curiosity, we refit the model given in Eq. 6, using only questions from the general block. Here, we used a question subtype predictor that indicated whether a question was a trivia question, or about a household tip (the two subtypes of question in the general block). Since this predictor is multi-colinear with usefulness judgements (as trivia questions are judged as less useful than household tips), we centered usefulness judgements within each question subtype. Thus, the effect of usefulness judgments on waiting in this model, as reported in the main text, is independent of the difference in usefulness between trivia questions and household tips.

Assessing the Effect of Prediction Errors on Subsequent Waiting

While our experimental design is constrained in providing causal support for the entire rational framework, it does provide causal support for parts of it. Specifically, we hypothesize in the main text that the energizing effect of motivation is due to waiting choices being influenced by the average value of information for a participant[9]. Influence of such an average value of information should also be evident on a trial-by-trial basis. If participants' choices are guided by such an average value, and that average is updated based on ongoing learning, we should be able to observe the updating process by assessing the influence of prediction errors (PE) on subsequent trials. While prediction errors in the waiting task are known to influence subsequent memory[1], their influence on subsequent information-seeking is as of yet unknown.

According to our model (Fig. 1a), expectations of value for each question are influenced both by the information given in the specific question, and by the average value of information. We measure these expectations using the usefulness ratings (Fig. 2b). Upon reading the answer, our participants report the actual experienced value of the answer in their satisfaction ratings. The difference between these satisfaction ratings and the usefulness ratings serves as our operationalization of a PE - the difference between value expectations before and after reading an answer. The rational theory would posit that large positive PEs should result in more waiting on the subsequent trial, since the average value of information should have been updated upwards, and vice versa for large negative PEs. Crucially, since the order of questions was fully randomized, finding any such effect would lend causal plausibility to the rational framework.

To test this hypothesis, we fit the following regression model to waiting choices:

waited ~ wait duration + usefulness + question type * previous satisfaction +

+ question type * previous usefulness +

+ (1 + duration + usefulness + question type * previous satisfaction +

+ question type * previous usefulness | participant) +

+ (1 + duration + previous satisfaction + previous usefulness | question)

Supplementary Equation 13

Importantly, both the previous usefulness ratings and the previous satisfaction ratings in this equation vary on a trial-by-trial basis, and so are adequate for assessing the trial-by-trial influence of PEs on waiting choices. While satisfaction was rated by the same participant making the waiting choices constituting the outcome measure for this model, usefulness ratings

in this equation, as in previous models, are the group average for the set of participants that rated each question. We are assuming that ratings elicited from other participants can predict waiting behavior, an assumption largely vindicated by our previous analyses using question usefulness. Moreover, each type of question - COVID-19 related questions and general questions - was administered in separate blocks of the waiting task, thus ensuring that any trial-by-trial effects are not confounded by question type.

For this model, the rational framework would predict a positive coefficient for previous satisfaction and a negative one for previous usefulness[10,11]. Together, this would constitute the difference between satisfaction and usefulness, or the PE, calibrated for different uses of the Likert scale for the two measures.

Assessing the Effect of Time

Our set of questions was most novel when we started data collection. As the knowledge about the pandemic spread throughout March and April, we expected curiosity and learning to change with time. To assess the effect of time on epistemic behavior, we fit the following multilevel logistic regression with time as a continuous predictor:

waited ~ wait duration + usefulness + question type * COVID-19 concern * date +

+ (1 + question type | participant) +

+ (1 + COVID-19 concern * date | question),

family = bernoulli() Supplementary Equation 14

satisfaction ~ usefulness + question type * COVID-19 concern * date +

+ (1 + question type | participant) +

+ (1 + COVID-19 concern * date | question),

family = ordered(link=”logit”) Supplementary Equation 15

recalled ~ usefulness + question type * COVID-19 concern * date +

+ (1 + question type | participant) +

+ (1 + COVID-19 concern * date | question),

family = bernoulli()   Supplementary Equation 16

Beyond an interest in the effect of time on information-seeking, the above models also serve as a control analysis. Since COVID-19 concern levels varied throughout the data-collection period, differences in information-seeking associated with COVID-19 concern may instead be related to differences between data collection days. Any effect of COVID-19 concern found in the model above would be evidence against this possibility, as it would be adjusted for any linear effect of time. To further control for any shape of time-based effect, and make sure our main findings generalize over all data-collection days, we also fit the following model, with collection day as a random factor:

waited ~ wait duration + usefulness + question type * COVID-19 concern +

+ (1 + wait duration + usefulness + question type | participant) +

+ (1 + wait duration + COVID-19 concern | question) +

+ (1 + wait duration + usefulness +

+ question type * COVID-19 concern | date)   Supplementary Equation 17

Assessing the Effect of Positive Affect

For completeness, we added positive affect, the third component to emerge from the BPCA analysis we conducted (see above), to the model predicting waiting from motivational states. The model can be described as follows:

waited ~ wait duration + usefulness + question type * COVID-19 concern +

+ question type * non-specific anxiety +

+ question type * positive affect +

+ (1 + wait duration + usefulness + question type | participant) +

+ (1 + wait duration + COVID-19 concern +

+ non-specific anxiety + positive affect | question)   Supplementary Equation 18

Assessing the Effect of Proportion of ‘Known’ Responses

In this study, we focused on how information-seeking is guided by informational utility. Another important determinant of information-seeking is the amount of information expected from an answer, depending on a participants’ existing knowledge about the question[12–15]. We did not design our experiment to provide a good measurement of the influence of existing knowledge on waiting behavior. Nonetheless, the proportion of questions each participant responded to as known can serve as an imperfect proxy for the confidence participants have in their knowledge regarding the type of questions presented in the experiment.

Since questions responded to as ‘Known’ did not contribute to our main index of information-seeking, one might hypothesize that differences in reporting ‘Known’ between our participants might bias our main results. That is not the case – while differences in reporting ‘Known’ alter the amount of data each participant contributes, and hence the variance of our estimates for each participant, it does not bias the main information-seeking index, which is defined as the proportion of ‘Wait’ vs. ‘Skip’ responses. Since throughout our analysis pipeline we used multilevel Bayesian regression models, differences in variance are fully adjusted for, as uncertainty is propagated and accounted for throughout the model[8].

We thus proceeded to test the effect of proportion ‘Known’ responses on information-seeking. First, we observed that the proportion of COVID-19-related questions and general questions reported as known was highly correlated $r=0.67$, $p<0.001$. Next, we entered proportion responded ‘Known’ for both types, standardized across participants, as a covariate in the model predicting waiting. The model can be described by the following equation:

waited ~ wait duration + COVID-19 concern *question type* proportion known +

+ usefulness * question type * proportion known +

+ (1 + wait duration + usefulness + question type | participant) +

+ (1 + wait duration + COVID-19 concern * proportion known | question)

Supplementary Equation 19

Since the relationship between confidence in prior knowledge and curiosity is often predicted and reported to be in the shape of an inverse U[12,14,16,17], we fit another model, allowing for proportion known to take a second order polynomial shape. An inverse-U relationship between confidence and curiosity would predict a negative coefficient for squared proportion known on waiting. The model can be described by the following equation:

waited ~ wait duration +

+ COVID-19 concern *question type* (proportion known + proportion known^2) +

+ usefulness * question type * (proportion known + proportion known^2) +

+ (1 + wait duration + usefulness + question type | participant) +

+ (1 + wait duration + COVID-19 concern *

* (proportion known + proportion known^2) | question)   Supplementary Equation 20

Supplementary Note 1

COVID-19 Concern and Non-Specific Anxiety

We found that ratings of COVID-19 concern were higher for participants who experienced job loss t(1069.84)=12.49, p< 0.001, a reduction in income t(3790.22)=12.26, p< 0.001, or self-isolation t(2129.50)=7.98, p< 0.001, relative to participants who did not experience these events (Fig. 5d). A similar pattern was observed for non-specific anxiety (job loss: t(930.69)=15.42, p< 0.001, income decrease: t(3448.08)=13.94, p< 0.001, self-isolation: t(2482.32)=5.42, p< 0.001; all degrees of freedom are given with the Welch correction for unequal variances).

We find a significant non-linear component in the development of COVID-19 concern over time SD=0.76, 95% Posterior Interval (PI) = [0.36,1.45], but not a linear increase b=1.15, 95% PI = [-0.23,2.42]. As can be seen in Fig. 5a, COVID-19 concern rose during the last two weeks of March, before plateauing and gradually decreasing. A similar non-linear component is found for non-specific anxiety SD=0.53, 95% PI = [0.16,1.14].

We observed a significant non-linear component in the change of COVID-19 concern with the social distancing measure SD=0.11, 95% PI = [0.02,0.28]. As can be seen in Fig. 5b, COVID-19 concern rose with social distancing behavior, and then plateaued. Between-state variance in COVID-19 concern levels was larger than zero SD=0.05, 95% PI = [0.02,0.08]. A non-linear component is significant also for non-specific anxiety SD=0.08, 95% PI = [0.003,0.31], which also varied considerably between states SD=0.06, 95% PI = [0.03,0.09].

Overall, COVID-19 concern and non-specific anxiety are moderately correlated r=0.44, p< 0.001. For completeness we also report their correlations with positive affect, the third component emergent from the BPCA analysis. COVID-19 concern is very weakly anticorrelated

with positive affect r=-0.08, p<0.001, while non-specific anxiety is robustly anticorrelated with positive affect r=-0.50, p<0.001.

Incentive Value of Questions

Waiting

Our findings reveal that information-seeking is cost-benefit rational (Supplementary Figure 2a): participants are more likely to wait for questions judged as useful, whether these are COVID-19-related or general questions b=0.66, 95% PI=[0.53,0.79]. As expected from a cost-benefit analysis, willingness to wait also diminishes for longer wait durations b=-0.07, 95% PI=[-0.07,-0.06].

In addition, we find no significant interaction between question type and duration b=-0.008, 95% PI = [-0.03,0.02], nor between duration and usefulness b=0.004, 95% PI = [-0.02,0.03], and question type and usefulness b=-0.03, 95% PI = [-0.16,0.11]. The three-way interaction is negligible as well b=-0.002, 95% PI = [-0.03,0.02].

Satisfaction

Like information-seeking, answer satisfaction increases with question usefulness judgments b=0.53, 95% PI=[0.42,0.65]. However, we find no significant effect of wait duration on satisfaction b=-0.008, 95% PI = [-0.03,0.01]. On average, satisfaction was lower for COVID-19-related answers than for general answers b=-0.17, 95% PI = [-0.29,-0.05]. The dependence of satisfaction on usefulness was lower for COVID-19-related answers than for general answers b=-0.12, 95% PI = [-0.24,-0.006]. All other interaction terms are negligible.

Memory

We found that participants better remembered answers to questions that were judged as highly useful b=0.42, 95% PI=[0.21,0.63] (Supplementary Figure 2c). We find no significant effect of wait duration on recall b=-0.01, 95% PI = [-0.04,0.01], nor of question type b=0.11, 95% PI = [-0.12,0.33]. All interaction terms are negligible.

Motivational Effects on Information-Seeking

Waiting

In addition to results reported in the main text, we find that among people with higher COVID-19 concern, choices regarding COVID-19-related questions tend to be more sensitive to usefulness, relative to choices regarding general questions, as is evidenced by a marginally

significant interaction between COVID-19 concern and question usefulness b=0.03, 95% PI=[-3.45e-04,0.06].

We also observe a significant interaction between non-specific anxiety and question type b=-0.05, 95% PI = [-0.08,-0.02], such that the decrease in waiting associated with higher non-specific anxiety is more pronounced for COVID-19-related questions. All other interactions involving affective concern are negligible.

Satisfaction

In addition to results reported in the main text, we find a significant interaction between COVID-19 concern and question usefulness b=0.05, 95% PI = [0.02,0.07], such that participants with higher COVID-19 concern produce satisfaction ratings that are more usefulness sensitive. We also find a small but significant interaction between non-specific anxiety and usefulness b=-0.04, 95% PI = [-0.07,-0.01], such that participants with high non-specific anxiety produce satisfaction ratings that are less usefulness sensitive. All other interactions involving motivational measures are negligible.

Memory

No other interaction is significant beyond the terms reported in the main text.

When adding the number of answers waited for as a covariate to this model, results are virtually unchanged. The number of answers waited for is not a significant predictor of memory, b=0.04, 95% PI = [-0.004, 0.08], with the sign of the coefficient opposite of that predicted by a list-length effect. Such an independence of memory from list-length has already been observed in studies of curiosity[1,14]. The effects of question usefulness, COVID-19 concern and non-specific anxiety on memory, as well as the interactions of these factors with question type, remain unchanged when adjusting for number of answers waited for.

The Effect of Motivational States on Usefulness Judgments

In addition to the results reported in the main text, we found that COVID-19-related questions were judged more useful than general questions b=0.61, 95% PI = [0.32,0.92]. Crucially, the effects of motivational state on usefulness judgements cannot be explained by a bias inducing participants to produce usefulness ratings that are congruent with waiting task choices, since usefulness judgments and waiting decisions were measured for different questions for each participant.

Results from the Joint Analysis of Waiting Choices and Usefulness Judgments

The indirect effect of COVID-19 concern on waiting choices, via usefulness judgements is reported in the main text, as is the percentage of the effect mediated by usefulness judgments. The 95% PI for this quantity includes values above 100% since the model indicates that suppression might exist between the three variables[18]. As detailed in Fig. 2d, the direct effect of COVID-19 concern on waiting choices is negligible in the joint analysis model, and could plausibly be negative b=0.06, 95% PI=[-0.07, 0.20].

Prediction Errors and Subsequent Waiting

In the main text we explain how PEs on trial n-1 influence waiting on trial n, such that participants are more likely to wait for answers after a large positive PE, and less likely to wait after a large negative PE (Fig. 4). The PE effect is captured by the coefficients for usefulness and satisfaction, as reported in the main text.

In addition to these main coefficients of interest, the two covariates added to the model (Supplementary Equation 12) significantly predicted waiting, as expected (wait duration b=-0.07, 95% PI = [-0.08, -0.06]; current question usefulness b=0.73, 95% PI = [0.55, 0.92]). There was no significant main effect of question type b=0.05 95% PI = [-0.13, 0.22]. The interaction terms between question type and previous satisfaction (b=0.09, 95% PI = [0.03, 0.14]) indicated that the relative weighting of satisfaction ratings in the PE term differed by question type. No significant interaction between question type and previous usefulness was observed (b=0.03, 95% PI = [-0.01, 0.07]).

The Effect of Time

The acute relevance of COVID-19 information to our participants was underscored by a reduction in waiting for COVID-19-related questions over the course of the 8 weeks of data collection; simple effect b=-0.09, 95% PI = [-0.17,-0.003]. Waiting for general questions over this period did not change significantly; simple effect b=0.001, 95% PI = [-0.07,0.07]. The interaction between question type and time of data collection is significant b=-0.04, 95% PI = [-0.07,-0.02]. No other effect or interaction involving time was significant, while the effect of COVID-19 concern and its interaction with question type were very similar to the ones reported in the main text.

A similar pattern emerged for satisfaction ratings. A significant interaction between date and question type indicated that COVID-19-related answers saw a selective decrease in satisfaction over the data collection period b=-0.07, 95% PI=[-0.10,-0.05]. No main effect of date was found b=0.01, 95% PI=[-0.04, 0.05]. COVID-19 concern exerted a similar main effect on satisfaction

and a similar interaction with question type as in our main analysis. The effect of COVID-19 concern on satisfaction did become stronger over the data collection period, as evidenced by the interaction with date b=0.06, 95% PI=[0.01, 0.10]. No other interaction with time was significant.

A significant interaction between date and question type was also found for memory, such that memory for COVID-19-related questions improved relative to general questions over the data collection period b=0.04, 95% PI=[0.01, 0.08]. The detrimental effect of COVID-19 concern on memory also strengthened over time, as evidenced by the interaction term b=-0.04, 95% PI=[-0.07, -0.0003]. No other effect of time was significant, and the main effect of COVID-19 concern and its interaction with question type were similar to those reported in the main analysis.

Lastly, the effects of COVID-19 concern and question type on waiting were unchanged also in the model treating time as a random factor (Supplementary Equation 15). Variability of the COVID-19 concern effect by data-collection date was negligible SD=0.05, 95% PI = [0.00, 0.17], as was the variability of the interaction effect between COVID-19 concern and question type SD=0.03, 95% PI = [0.00, 0.07] (for comparison, day-to-day variability in average waiting was SD=0.21, 95% PI=[0.11, 0.34]). Thus, our main findings generalize well across data-collection days.

The Effect of Positive Affect

In a model together with COVID-19 concern and non-specific anxiety (Supplementary Equation 18), positive affect did not significantly predict waiting b=0.02, 95% PI = [-0.07, 0.11]. Nor was there a significant interaction between positive affect and question type in predicting waiting b=-0.02 95% PI = [-0.06, 0.02]. The effects of COVID-19 concern and non-specific anxiety, as well as the interactions of these motivational states with question type remained quantitatively and qualitatively similar even when adjusting for positive affect.

The Effect of Proportion of 'Known' Responses

Adding proportion known as a covariate to the model predicting waiting from COVID-19 concern, question usefulness, and wait duration (Supplementary Equation 19) did not alter any of the inferences reported in the main text. We observed that participants who tended to respond 'Known' more often tended to wait less for answers, as evidenced by a main effect of proportion known on waiting b=-0.32, 95% PI=[-0.39, -0.25]. This pattern undermines a possible alternative explanation to the effect of COVID-19 concern on waiting – that people who are

concerned about COVID-19 know more about it, and merely by virtue of their knowledge seek more information.

The effect of proportion ‘Known’ responses was stronger for general questions than COVID-19-related questions, interaction b=0.10, 95% PI = [0.04, 0.16]. Furthermore, we observed that with a higher proportion of ‘Known’ responses the effect of question usefulness on waiting is attenuated, as evidenced by the two-way interaction term b=-0.18, 95% PI = [-0.23, -0.12]. This effect was stronger for general questions, as indicated by a significant three-way interaction with usefulness and question type b=0.06, 95% PI = [0.01, 0.12]. The three-way interaction between proportion ‘Known’ responses, COVID-19 concern and question type was also significant b=-0.04, 95% PI = [-0.08, -0.01]. This three-way interaction is manifested by proportion ‘Known’ associated with a significant attenuation of the directing effect of COVID-19 concern, that is the effect of COVID-19 concern on COVID-19-related questions, b=-0.10, 95% PI = [-0.16, -0.03]. No significant attenuation is found for general questions, meaning that the energizing effect is not attenuated by proportion ‘Known’ b=-0.01, 95% PI = [-0.08, 0.06]. Consult Supplementary Figure 4 for a clear visual representation of these patterns.

Lastly, we do not find an inverse-U-shaped effect of proportion ‘Known’ on waiting – when adding proportion ‘Known’ squared to the model (Supplementary Equation 20), the coefficient is estimated to be positive rather than negative, contrary to the prediction b=0.10, 95% PI = [0.03, 0.17]. The addition of this term modulates the reduction in waiting with higher proportion ‘Known’ but does not change the fact that waiting monotonically decreases with proportion ‘Known’.

Supplementary Note 2

In addition to the experiment described above and in the main text, we conducted a supplementary experiment in a smaller sample with self-report ratings of curiosity rather than waiting task choices as the main dependent measure. The use of curiosity ratings also allowed us to use a larger variety of questions, including questions we did not have the answer to. Additionally, since answers were not displayed in this experiment, the theoretical confounding phenomenon of directional transfer, by which learning during the waiting task affects subsequent responses on the affective questionnaires, is rendered highly improbable.

This experiment thus tests whether our main conclusions generalize to a second measurement modality, over a more variable question set, and with minimized learning during the waiting task.

Participants

200 new participants (ages 18-71) were recruited through MTurk on April 14, 2020. Participants were paid $2.30 for participation, plus a $2 bonus for attentive completion of the experiment.

Stimuli

A set of 70 questions was assembled for this experiment, half of them COVID-19-related, half general questions. Across these two categories, half of the questions constituted a subset of items randomly sampled from the main experiment - which we denote as *answerable* questions. Some of these items were adapted to reflect the changes in officially sanctioned knowledge regarding COVID-19. The rest of the questions were composed such that an answer was not yet known, or would probably never be known (e.g., *Will the career of Golden State Warriors star Steph Curry last at least 10 more years?, Who was the first COVID-19 patient in the US?*) - these are *unanswerable* questions.

For each participant, 10 COVID-19-related questions and 10 general questions were set aside for the usefulness rating task, with the rest used for the curiosity rating task.

Procedure

The experimental procedure was identical to session 1 of the main experiment, bar the replacement of the waiting task with a curiosity rating task. On each trial of this task, participants were presented with a question and asked to rate how curious they were to know the answer to the question on a scale of 1-7. No answers were given, and hence answer satisfaction was not measured.

Participants also rated questions on their confidence in knowing the answer (again on a 1-7 scale). Half of participants rated each question both on curiosity and then on confidence (in this order). The other group first rated half of the questions on curiosity, and then the other half on confidence. Confidence ratings are not analyzed for this paper, and no difference was found between the two groups.

Statistical Analysis

Data from 11 participants (5.50%) who reported less than perfect English fluency were excluded from analysis.

To evaluate whether the results from the main experiment replicate when curiosity is measured by self-report, the following multilevel ordered-logistic regression model was fit to the data:

curiosity ~ answerability + question type * COVID-19 concern * usefulness +

+ question type * non-specific anxiety * usefulness +

+ (1 + answerability + question type | participant) +

+ (1 + COVID-19 concern + non-specific anxiety),

family = ordered(link = "logit") Supplementary Equation 21

Where *question type*, as in previous models, has two levels: COVID-19-related or general. The COVID-19 concern and non-specific anxiety measures were made up from the same lists of items used for analysis of the main experiment. Question usefulness estimates were computed using an IRT model as described in the methods section for the main experiment above.

Results

Self-reported curiosity, with the expanded questions set, replicates the findings from the main experiment (Supplementary Figure 6). Usefulness judgements were a robust predictor of curiosity ratings b=0.66, 95% PI=[0.46,0.85]. Higher COVID-19 concern was associated with higher curiosity in general b=0.79, 95% PI=[0.55,1.02], but especially so for COVID-19-related questions, as evidenced by a significant interaction of COVID-19 concern and question type b=0.15, 95% PI=[0.05,0.26]. Higher non-specific anxiety was associated with lower overall curiosity b=-0.35, 95% PI=[-0.59,-0.12].

Additional to these effects, which constitute a conceptual replication of the main experiment, we find a significant interaction between usefulness and question type, such that participants exhibited lesser sensitivity to judged usefulness in curiosity ratings of COVID-19-related questions relative to general questions b=-0.21, 95% PI=[-0.38,-0.03]. An interaction between non-specific anxiety and question type is also evident b=-0.18, 95% PI=[-0.28,-0.08], as is an interaction between non-specific anxiety and judged usefulness, such that people with higher non-specific anxiety produce curiosity ratings that are less sensitive to judged usefulness b=-0.14, 95% PI=[-0.26,-0.02]. This is true especially for general questions, as evidenced by a significant three-way interaction between non-specific anxiety, judged usefulness and question type b=0.10, 95% PI=[0.010,0.19]. All other effects were negligible.

Figures

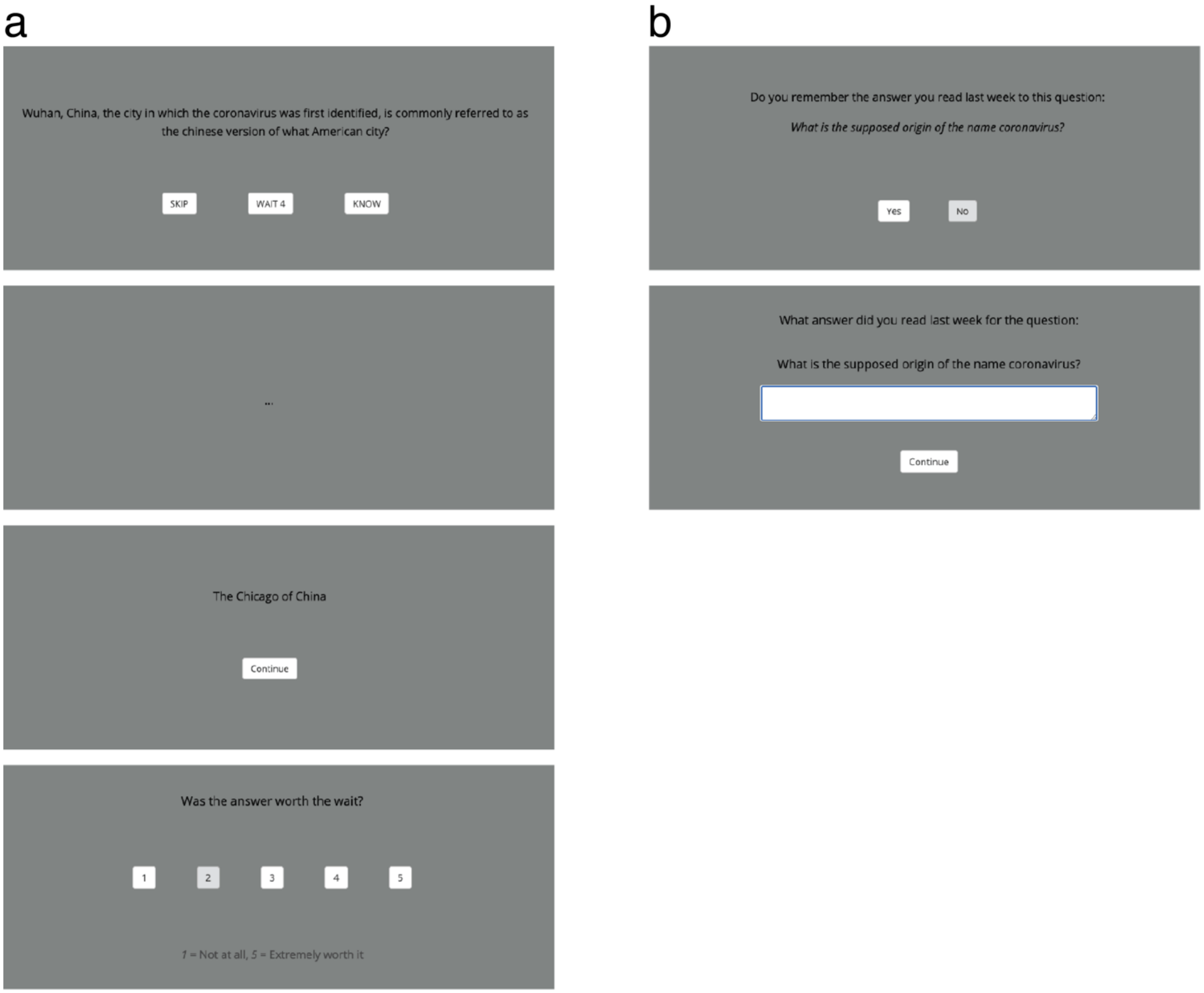

Supplementary Figure 1. Screenshots of tasks in the main experiment. On each trial of the waiting task (a), participants were presented with a question, and the three choice buttons depicted in the top panel. The number on the WAIT choice button denoted the number of seconds they were required to wait in order to view the answer (drawn from the set 4/8/12/16s). If participants chose to wait, an ellipsis slide was presented for the denoted duration, followed by the answer. After confirming they had read the answer, participants rated their satisfaction with the answer on a Likert scale. One each trial of the answer recollection task (b), conducted 7 or 8 days after session 1, participants were presented with a question they had chosen to wait for. Participants indicated whether they remembered the answer to the question, and if so, they typed their recalled answer in a text box.

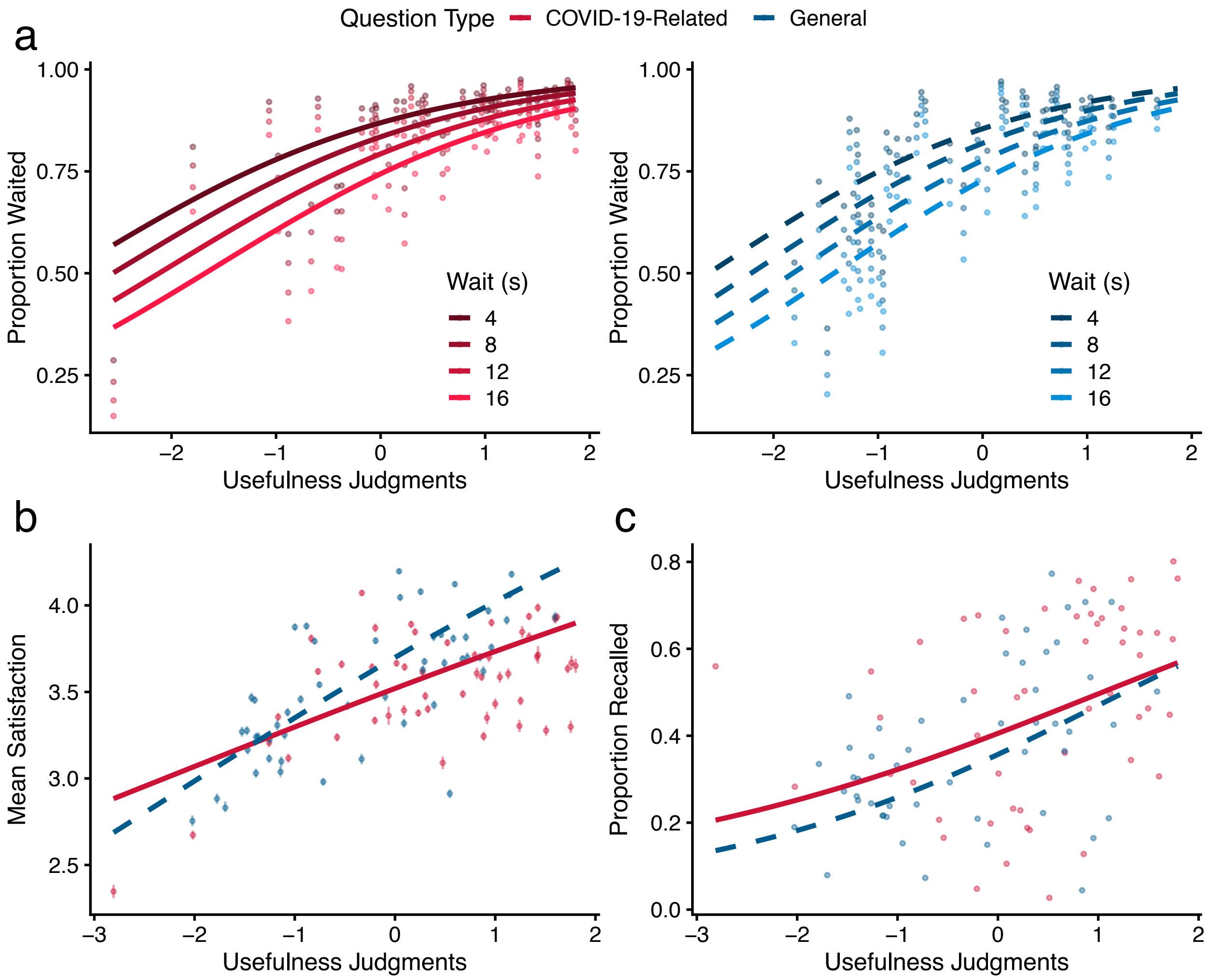

Supplementary Figure 2. The incentive value of questions is predicted by usefulness judgments. (**a**) Participants were more likely to wait for questions judged as more useful, and less likely to choose to wait long durations. These effects hold for both COVID-19-related questions (left) and general questions (right). Data presented as marginal predictions per question from a multilevel logistic regression model, lines denote the median prediction from the model. **(b)** Judged question usefulness is also positively correlated with self-reported satisfaction with answers. Data presented as mean values ±1 SEM, lines denote median prediction from a multilevel ordered-logistic regression model; n=5088 participants. **(c)** Judged usefulness is associated with better memory for the answer, assessed with a memory test one week after the waiting task. Data presented as marginal means, lines denote median prediction from a multilevel logistic regression model. Source data are provided as Source Data files.

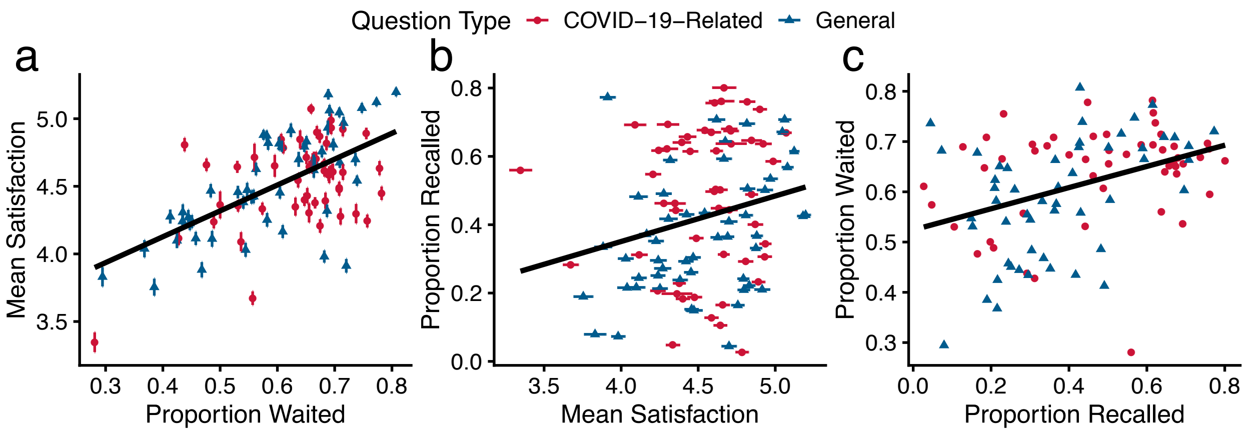

Figure. S3. Participants tend to seek information that on average would make them more satisfied, and that they are more likely to remember. (a) Questions for which a high proportion of participants chose to wait tend to have satisfying answers, and vice versa r=0.60, n=104, two-sided p=1.26×10-11. Data presented as mean values ±1 SEM; n=5088 for satisfaction error bars. (b) Satisfaction with answers is correlated with probability of recalling them r=0.23, n=104, two-sided p=0.02. Data presented as mean values ±1 SEM; n=5088 for satisfaction error bars. (c) Proportion of participants waiting for a question is correlated with the proportion of participants recalling the answer to said question r=0.39, n=104, two-sided p=4.85×10-5. Linear regression lines are plotted for visualization purposes. p-values were not corrected for multiple comparisons. Source data are provided as Source Data files.

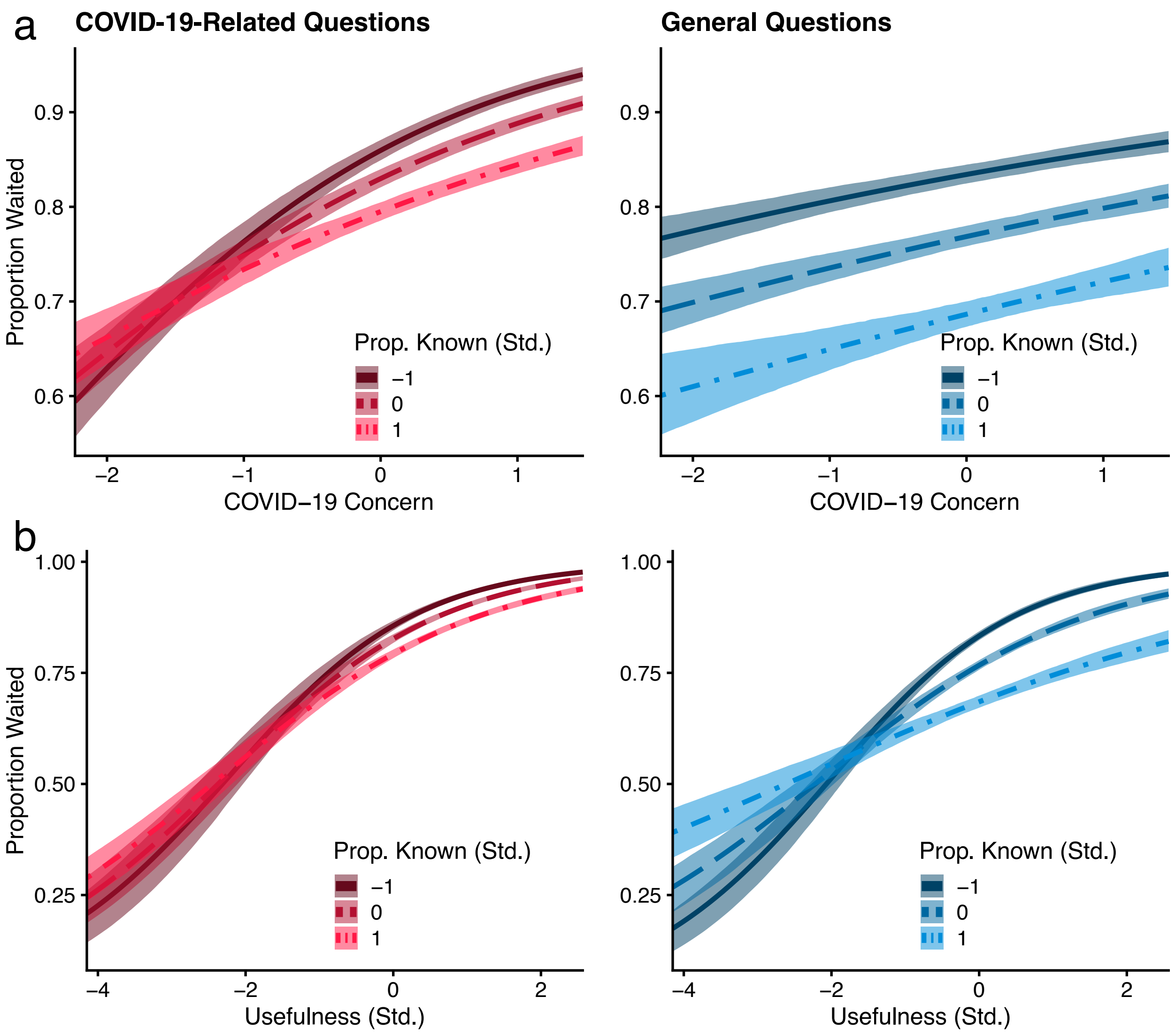

Supplementary Figure 4. The effects of self-reported knowledge on waiting choices (a) Participants who responded to a greater proportion of questions as known tend to wait less for answers overall. Among such participants the directing effect of COVID-19 concern is attenuated: COVID-19 concern is a weaker predictor of waiting for COVID-19-related question (left panel). The energizing effect of COVID-19 concern is unaffected: COVID-19 concern predicts a rise in waiting for general questions regardless of the proportion of known answers (right panel). (b) Among participants who responded to a greater proportion of questions as known, question usefulness is a weaker predictor of waiting choices for both questions type, but especially so for general questions (right panel). Lines denote median posterior predictions, shaded areas mark 50% PIs. Source data are provided as Source Data files.

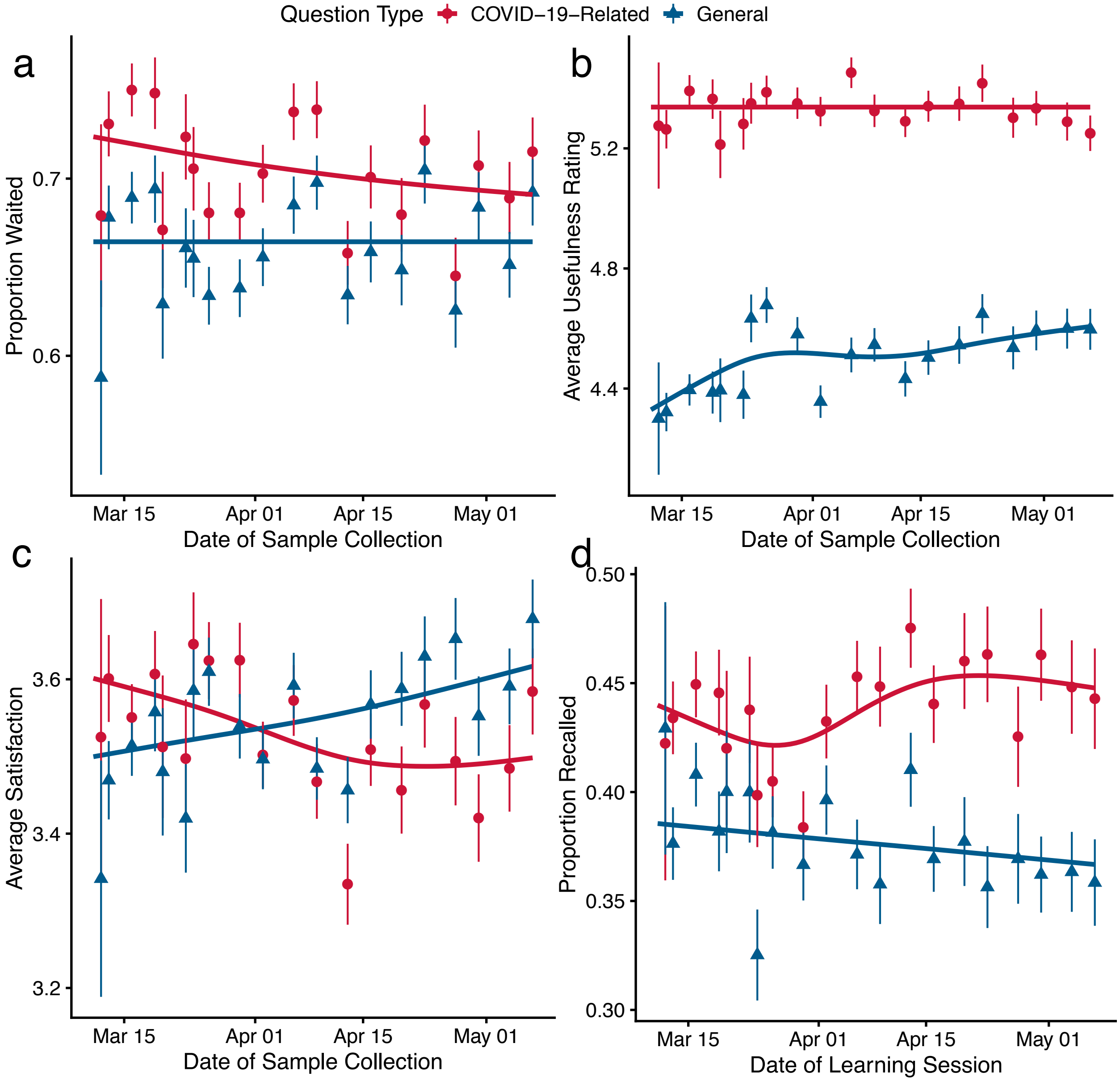

Supplementary Figure 5. Timelines of epistemic behavior (a) The change in proportion of answers waited for during the data collection period. n=5325 participants. (b) The change in average usefulness judgments during data collection. n=5375 participants. (c) The change in average satisfaction during data collection. n=5087 participants. (d) The changes in proportion of recalled answers during data collection, plotted by the date of the first learning session, for easy comparison with the other variables. n=3772 participants. Data presented as mean values ±1 SEM, lines denote a general additive model fit to participant-wise averages. Source data are provided as Source Data files.

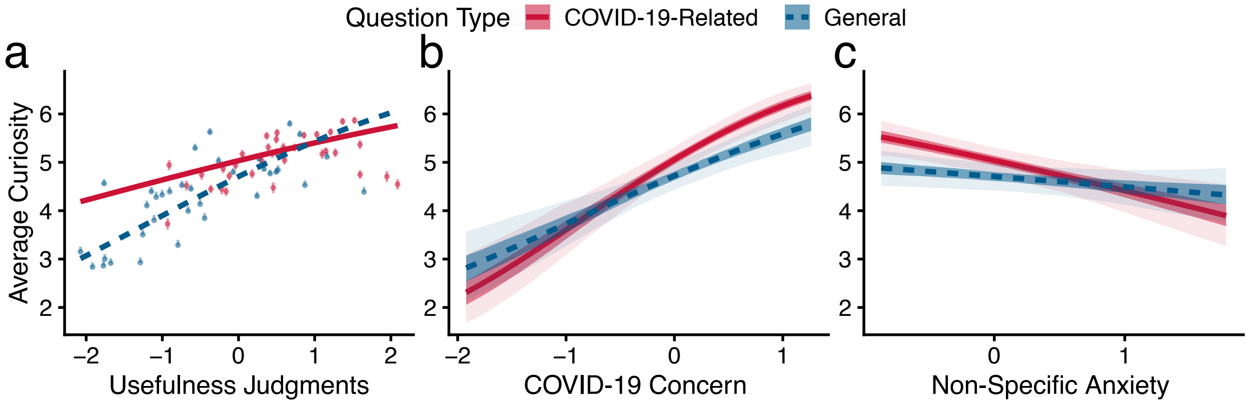

Supplementary Figure 6. Curiosity self-reports collected in the supplementary experiment replicate our main findings. (a) Usefulness judgements predict curiosity towards both COVID-19-related questions, and general questions, though more so for the latter. n=189 participants. (b) High COVID-19 concern predicts high self-reported curiosity, especially so for COVID-19-related questions. (c) Higher non-specific anxiety is associated with lower curiosity self-report levels. Lines denote mean posterior prediction; dark shaded areas mark 50% PIs, and light areas 95% PIs. Dots denote mean values with error bars spanning ±1 SEM. Source data are provided as Source Data files.

Supplementary Table 1.

**Type of coefficient**	**Prior**
Intercept (not applicable for ordered-logistic models)	Normal(0,1)
Group-level effects of predictors	Normal(0,1)
Scale of by-participant, by-question or by-state terms	Normal(0,1)
Correlation matrices for by-participant, by-question or by-state terms	LKJ(2)

Regularizing priors used in all regression models reported in this paper. Prior distributions are given in Stan syntax. All predictors used in models were centered and scaled prior to fitting, so that the same priors can apply to all parameters.

Supplementary Table 2.

Question type	Question	Answer	Standardized usefulness Estimate	Proportion waited	Mean satisfaction	Proportion recalled
General	What animal is thought to be immune to most all diseases, including cancer?	Shark	-0.02	0.81	5.20	0.85
General	What vegetable can prevent your windshield from freezing over in winter?	Potatoes: rub a cut potato over your windshield before a frost to prevent freezing	0.50	0.77	5.12	0.80
General	What popular snack food is effective to use as kindling to start a fire?	Doritos	0.18	0.75	5.08	0.87
General	In 1908, New York City passed the Sullivan Ordinance which made it illegal for women to do what in public?	Smoke	-0.77	0.74	4.54	0.87
General	What fruit has the highest content of vitamin C?	Camu Camu, a cherry-like fruit from Brazil	0.74	0.74	4.70	0.16

General	What's a simple trick for telling whether you're out of something when you are grocery shopping?	Take a picture of your fridge before leaving the house	0.45	0.72	3.91	0.93
General	How can you tell if a battery is dead without using any tools?	Drop it from a 3-inch height. If it bounces, it's dead.	0.81	0.72	4.97	0.80
General	What salad dressing ingredient is also an efficient weed killer?	White vinegar	0.21	0.71	4.68	0.94
General	What tropical fruit can be regrown from its scraps?	Pineapple, by planting the green leaves	-0.01	0.71	5.05	0.93
General	How can you tell if an egg is cooked without breaking it?	Roll it along its long side. If it stops immediately, it's raw.	0.49	0.70	4.81	0.67
General	What item that can be found in any car can help you extract your car from mud or snow?	Floor mats - put them under the wheels for traction	1.01	0.69	5.06	0.95
General	In addition to chocolate, what other foods can be very harmful to dogs?	Grapes or raisins, onions, avocado	1.03	0.69	5.18	0.83

General	How do you put out a grease fire?	Deprive it of oxygen by covering, pouring baking soda if the fire is small, or using a fire extinguisher if it's large.	1.44	0.69	4.93	0.84
General	How do you make your smile look more genuine in photographs?	Squint your eyes	0.02	0.69	4.32	0.88
General	What's been released five times a year from January 1999 to fall 2008?	State quarters	-0.74	0.68	3.98	0.64
General	How far in advance of a trip is the most cost-effective time to purchase a plane ticket?	Between 105 and 54 days before the scheduled trip.	0.84	0.68	4.76	0.31
General	How can you cool beverages more quickly?	Wrap a wet paper towel around the bottle and put in the fridge	0.40	0.67	4.67	0.86
General	Where in the supermarket can you usually find the cheaper items?	Below eye level	0.77	0.66	4.62	0.87

General	What can you use instead of fabric softener to soften sheets and remove odors?	White vinegar	0.57	0.66	4.69	0.82
General	While tomato juice probably will not work, what does work to remove skunk odor?	Dish soap, peroxide, and baking soda	0.37	0.65	4.83	0.67
General	In 1568 the Catholic Church sentenced the entire population of which nation to death as heretics?	The Netherlands	-0.82	0.65	4.79	0.64
General	How to chop an onion without crying?	Freeze before chopping, or hold a piece of bread in your mouth, half sticking out	0.62	0.64	4.70	0.79
General	How much money should I keep as an emergency fund?	At least $2,467 according to current research	0.99	0.62	4.91	0.40
General	The title of the song ""Kumbaya"" is actually a distortion of what three-word phrase?	Come by her	-1.45	0.61	4.17	0.78

General	When is the next total solar eclipse visible in North America projected to occur?	April 8th, 2024	-0.24	0.61	4.80	0.63
General	What album spent a record 741 weeks on Billboard's Top 200 Albums chart?	Dark Side of the Moon	-1.38	0.60	4.45	0.83
General	What's a better investment: stocks or real estate?	Over the past 150 years, real estate, if you rent it out	0.60	0.60	4.82	0.91
General	How can you soften brown sugar that has hardened into a lump?	Microwave next to a glass of water	0.30	0.58	4.82	0.84
General	What was put in place by the Ancient Greeks before and during all Olympic festivals?	A truce	-1.10	0.58	4.25	0.79
General	Who beat out FDR and Gandhi to be named Time's Person of the Century in 1999?	Albert Einstein	-1.00	0.58	4.87	0.84

General	Which country has the most public libraries?	Russia	-0.89	0.58	4.88	0.69
General	What's an easy way to waterproof canvas shoes?	Spread beeswax over them and then blow dry until you can't see the beeswax anymore	0.20	0.56	4.63	0.75
General	What can you do to make dry beans cook faster?	Add baking soda, soak in advance, use a pressure cooker	0.30	0.56	4.42	0.88
General	The apgar score is used to measure the color and condition of what?	Newborns	-0.51	0.55	4.42	0.83
General	How can people under 25 rent a car without paying extra fees?	Sign up for a free USAA membership	-0.15	0.55	4.47	0.48
General	What Indianapolis grocer made his name by selling canned pork and beans to U.S. troops during the Civil War?	Gilbert Van Camp	-1.37	0.54	4.03	0.80

General	Which was the only one of his sculptures that Michelangelo signed?	The Pieta	-1.07	0.54	4.38	0.75
General	Which Yellowstone geyser, with eruptions 100 fee taller than Old Faithful's, is the world's tallest?	Steamboat Geyser	-0.95	0.53	4.46	0.79
General	How can you give yourself a layered haircut?	Tie your hair into a tight ponytail at the front of your head and cut it.	-0.38	0.49	4.11	0.85
General	What common aquarium fish is named for the naturalist who discovered it in Trinidad in 1876?	The guppy	-1.41	0.48	4.47	0.87
General	What term does rapper B.G. say he wishes he'd patented when he used it for the title of his massive 1999 hit?	Bling bling	-1.74	0.47	3.88	0.91
General	In what city could you see the Seagull Monument at Temple Square?	Salt Lake City	-1.25	0.46	4.11	0.83

General	What is the name of the ship that carried Captain Cook to Australia?	Endeavour	-1.37	0.45	4.24	0.79
General	What name is shared by the rivers that run through Denison, TX and Hanoi, Vietnam?	Red River	-1.24	0.45	4.23	0.88
General	What famed TV personality wrote the screenplay for 1968's Planet of the Apes?	Rod Serling	-1.50	0.44	4.27	0.78
General	Which of the four biblical Gospels was written by a tax collector?	Matthew	-1.17	0.43	4.31	0.81
General	Who christened Hawaii ""the Sandwich Islands"" in 1778?	Captain Cook	-1.35	0.43	4.24	0.89
General	Who discovered the Victoria Falls?	David Livingstone	-1.13	0.42	4.10	0.76
General	Who's the only two-time Sports Illustrated Sportsman of the Year, so named in 1996 and 2000?	Tiger Woods	-1.45	0.41	4.28	0.94

General	What Canadian singer appeared on the first cover of Entertainment Weekly?	K.D. Lang	-1.97	0.39	3.75	0.64
General	Who wrote ""Who's Afraid of Virginia Woolf?""	Edward Albee	-1.14	0.37	4.04	0.79
General	In 1991, who finally broke Bob Beamon's decades-old long jump record?	Mike Powell	-1.66	0.29	3.83	0.64
COVID-19-related	What did 75% of the people who have died from the new coronavirus have?	Pre-existing health conditions, including cardiovascular diseases and diabetes.	1.12	0.78	4.45	0.84
COVID-19-related	How long does the new coronavirus survive on surfaces?	Studies suggest that coronaviruses (including preliminary information on the new virus) may persist on surfaces for a few hours or up to several days.	1.56	0.78	4.63	0.74

COVID-19-related	What is the source of coronavirus in humans?	Animals, probably bats	0.77	0.76	4.24	0.89
COVID-19-related	At the end of February, Amazon banned over a million products because of what?	They falsely claimed to cure or prevent coronavirus	0.09	0.76	4.89	0.41
COVID-19-related	What is the most effective measure to prevent infection with the coronavirus?	Washing your hands frequently	1.59	0.74	4.30	0.75
COVID-19-related	Why does the CDC currently recommend that healthy people do not wear N95 face masks?	Reserves need to be saved for people who are infected and for healthcare professionals.	1.00	0.71	4.60	0.74
COVID-19-related	How long is the incubation period for the new coronavirus?	The incubation period - the time between catching the virus and beginning to have symptoms of the disease - is estimated to range from 1-14 days, most commonly around five days.	1.44	0.71	4.92	0.82

COVID-19-related	How soon is a vaccine for the new coronavirus likely to be available?	Not for at least several months from now	1.36	0.71	4.28	0.75
COVID-19-related	What sort of specimen is needed to test for the new coronavirus?	Nasal or oral swab or a sample of sputum	0.57	0.71	4.49	0.74
COVID-19-related	Why is the virus believed to have zoonotic origin, i.e., to have potentially come from animals?	The initial cases of the disease are thought to have been among people with a connection to a seafood wholesale market, which also sold live animals	0.22	0.71	4.48	0.44
COVID-19-related	An epidemic is the outbreak of a disease that affects many people at the same time. What does pandemic mean?	An epidemic that has spread around the world	0.71	0.70	4.61	0.98
COVID-19-related	Can the new coronavirus be transmitted by touching a soft surface like carpets or fabric?	Soft surfaces are less likely to transmit the virus than hard surfaces.	1.46	0.69	4.93	0.43

COVID-19-related	Wuhan, China, the city in which the coronavirus was first identified, is commonly referred to as the Chinese version of what American city?	The Chicago of China	-0.79	0.69	4.62	0.83
COVID-19-related	Can the new coronavirus be transmitted on a package from an infected country?	The virus lives for up to a few days on surfaces, and so packages cannot transmit the virus.	1.28	0.69	4.99	0.80
COVID-19-related	What are current Centers for Disease Control (CDC) guidelines for releasing someone from isolation?	Free of fever; no other symptoms, including cough; negative tests on at least two consecutive respiratory specimens at least 24 hours apart.	1.20	0.69	4.94	0.69
COVID-19-related	Approximately 80% of deaths attributed to the new coronavirus were among people of what age?	People over age 60	0.92	0.69	4.59	0.92
COVID-19-related	Which drug, which has been used as a treatment for the Ebola and	Remdesivir	0.75	0.69	4.59	0.42

	Marburg viruses, might be promising as a treatment for this new coronavirus?					
COVID-19-related	Can the new coronavirus be transmitted through mosquito bites?	The virus spreads through coughs and sneezes, there are no reports of transmission through mosquito bites.	1.20	0.69	4.82	0.97
COVID-19-related	Why is the new coronavirus called ""COVID-19""?	It stands for ""COronaVIrus Disease 2019""	0.02	0.68	4.39	0.88
COVID-19-related	What other animals have been found to have viruses with highly similar genome sequences as the current coronavirus?	Bats and pangolins	0.20	0.68	4.61	0.89
COVID-19-related	How many pandemics were there in the 20th century?	Three	-0.05	0.68	4.67	0.61
COVID-19-related	According to media reports, which 2011 movie has seen a large	Contagion	-1.25	0.67	4.21	0.79

	increase in popularity in the wake of the new coronavirus?					
COVID-19-related	What is the supposed origin of the name coronavirus?	The viruses have spikes on their outer surface that look like a crown (or corona in Latin)	-0.25	0.67	4.87	0.70
COVID-19-related	Does sleep make you more resistant to coronavirus infections?	Yes, by boosting the immune system	0.84	0.67	4.90	0.91
COVID-19-related	Can the coronavirus be transmitted through sexual intercourse?	The virus is probably not sexually transmissible, but infection does occurs when kissing.	0.82	0.67	4.70	0.91
COVID-19-related	What is the name of the repository of drugs and supplies the U.S. government is supposed to maintain in case of a public health emergency?	Strategic National Stockpile	0.16	0.67	4.38	0.81

COVID-19-related	The CDC recommend people wear cloth masks in what situations?	In public settings where other social distancing measures are difficult to maintain (e.g., grocery stores and pharmacies).	1.27	0.66	4.70	0.71
COVID-19-related	What are the most common symptoms of the new coronavirus?	Fever, cough, shortness of breath.	1.60	0.66	4.67	0.97
COVID-19-related	Why are N95 respirator face masks so named?	They are able to filter out at least 95% of airborne particles	-0.37	0.66	5.07	0.94
COVID-19-related	The name for the new disease COVID-19 adheres to WHO best practices in disease naming, which include guidelines to not name disease after what?	Locations, people, or animals	-0.28	0.66	4.64	0.90
COVID-19-related	Are the new coronavirus symptoms more similar to the symptoms of flu, or of seasonal allergies?	More similar to the symptoms of the ful	1.11	0.66	4.30	0.82

COVID-19-related	Are hand dryers effective in killing the new coronavirus?	No. To protect yourself against the new coronavirus washing your hands is the best preventive measure.	0.88	0.65	4.43	0.97
COVID-19-related	Can regularly rinsing your nose with saline help prevent infection with the new coronavirus?	No. There is no evidence that regularly rinsing the nose with saline has protected people from infection with the new coronavirus.	0.69	0.65	4.71	0.91
COVID-19-related	What is the name of the process health authorities use to understand the sources of infection and to prevent further transmission?	Contact tracing	0.25	0.65	4.40	0.58
COVID-19-related	Are antibiotics effective in preventing and treating the new coronavirus?	No, antibiotics do not work against viruses, only bacteria.	1.13	0.64	4.85	0.91
COVID-19-related	The first US death from the new coronavirus occurred in which state?	Washington	-0.24	0.64	4.54	0.83

COVID-19-related	It is recommended that healthcare professionals who may be working in contact with people with the virus wear what, in addition to personal protective equipment on their hands and bodies?	Approved filtering facemask respirators, such as the N95 respirator.	0.80	0.63	4.35	0.89
COVID-19-related	In the early stages of the new coronavirus, the number of new cases doubled approximately every how many days?	7.5 days	0.43	0.61	4.78	0.06
COVID-19-related	The official name of the new coronavirus is SARS-CoV-2. What does SARS stand for?	Severe acute respiratory syndrome	0.13	0.61	4.85	0.93
COVID-19-related	Currently, people who have traveled to a high-risk area or who may have been in contact with someone with the new coronavirus are recommended to do what?	Quarantine themselves for 14 days to make sure they are not infected.	1.63	0.60	4.65	0.89

COVID-19-related	When was the molecular structure of the coronavirus identified?	February 1st, 2020	-0.25	0.57	4.33	0.32
COVID-19-related	Does the new coronavirus affect older people, or are younger people also susceptible?	People of all ages can be infected by the new coronavirus. Older people appear to be more vulnerable to becoming severely ill with the virus.	1.28	0.56	4.71	0.84
COVID-19-related	Which celebrity said about the new coronavirus ""I've already been in this movie""?	Gwyneth Paltrow	-1.96	0.56	3.67	0.75
COVID-19-related	Can eating garlic help prevent infection with the new coronavirus?	There is no evidence from the current outbreak that eating garlic has protected people from the new coronavirus.	0.39	0.54	4.09	0.93
COVID-19-related	Which was the first US University to cancel classes because of the new coronavirus?	University of Washington	-1.16	0.53	4.36	0.84

COVID-19-related	On what date did the World Health Organization declare the new coronavirus outbreak to be a Public Health Emergency of International Concern?	January 30, 2020	0.03	0.53	4.64	1.00
COVID-19-related	What is the name of the province in China in which this new coronavirus was first discovered?	Hubei	-0.12	0.50	4.36	0.34
COVID-19-related	What is the name of the county in Italy that had the first coronavirus outbreak?	Lombardy	-0.61	0.49	4.24	0.68
COVID-19-related	What is the population of Wuhan, the city in which coronavirus was first identified?	11 million people	-0.56	0.48	4.66	0.44
COVID-19-related	In May 2018, Rear Admiral Timothy Ziemer left the White House staff and was not replaced. What was Rear Adm. Ziemer's responsibility in the White House?	Rear Adm. Ziemer led the global health security team, responsible for leading the US response to pandemics.	-0.85	0.44	4.81	0.82

COVID-19-related	What was the name of the British cruise ship that was quarantined in Japanese waters?	Diamond Princess	-1.06	0.43	4.12	0.79
COVID-19-related	What color latex gloves did Naomi Campbell wear to protect herself from coronavirus?	Pink	-2.71	0.28	3.35	0.81

Question and answer stimuli used in the main experiment, along with their mean usefulness judgment, proportion of participants waiting for the answer, mean satisfaction rating and proportion of participants correctly recalling the answer.

Supplementary Table 3.

Item	Response range	Correlation with scale mean excluding item
I am tense	1-4	0.72
I feel upset	1-4	0.69
I am worried	1-4	0.75
How anxious are you right now?	1-5	0.77
How anxious do you feel in general?	1-5	0.69
Did you experience anger during a lot of the day yesterday?	Yes/No	0.47
Did you experience depression during a lot of the day yesterday?	Yes/No	0.65
Did you experience sadness during a lot of the day yesterday?	Yes/No	0.64
Did you experience stress during a lot of the day yesterday?	Yes/No	0.60
Did you experience worry during a lot of the day yesterday?	Yes/No	0.60

Items of the non-specific anxiety scale, along with the response range for the items, and the correlation of each item scores with the mean score of the rest of the items. Cronbach's α for the scale is 0.90.

Supplementary Table 4.

**Item**	**Response range**	**Correlation with scale mean excluding item**
What do you think the chances are that you will personally be infected with the new coronavirus?	0-100%	0.40
What do you think the chances are that you will lose a loved one to the new coronavirus?	0-100%	0.41
In the next few weeks, how much will the lives of people around you will change as a result of the new coronavirus epidemic?	1-5	0.57
How anxious do you feel about the possibility of losing your life to the new coronavirus?	1-5	0.63
In the next few weeks, how much will your personal life change as a result of the new coronavirus epidemic?	1-5	0.64
In the next few weeks, how much will society change as a result of the new coronavirus epidemic?	1-5	0.51
How upset would you be if you find out that a close family member is infected with the new coronavirus?	1-5	0.45
How upset would you be if you find out that you are infected with the new coronavirus?	1-5	0.51
How worried are you about being infected with the coronavirus?	1-5	0.68
How much has your personal life changed relative to a month ago as a result of the new coronavirus epidemic?	1-5	0.54

How anxious do you feel about the following statements regarding the epidemic:		
Severe constraints have been placed on everyday life due to the epidemic	1-5	0.52
Economic prospects have become worse due to the epidemic	1-5	0.49
It is unclear when the state of emergency will be over	1-5	0.60
Social distancing might increase feelings of loneliness	1-5	0.49
The number of infected cases in the US keeps rising	1-5	0.66

Items of the COVID-19 concern scale, along with the response range for the items, and the correlation of each item scores with the mean score of the rest of the items. Cronbach's α for the scale is 0.88.

Supplementary References

1. Marvin, C. B. & Shohamy, D. Curiosity and reward: Valence predicts choice and information prediction errors enhance learning. *J. Exp. Psychol. Gen.* **145**, 266–272 (2016).
2. Kashdan, T. B. *et al.* The five-dimensional curiosity scale: Capturing the bandwidth of curiosity and identifying four unique subgroups of curious people. *J. Res. Pers.* **73**, 130–149 (2018).
3. Gartland, D., Bond, L., Olsson, C. A., Buzwell, S. & Sawyer, S. M. Development of a multi-dimensional measure of resilience in adolescents: the Adolescent Resilience Questionnaire. *BMC Med. Res. Methodol.* **11**, 134 (2011).
4. Tannenbaum, D., Fox, C. R. & Ülkümen, G. Judgment extremity and accuracy under epistemic vs. aleatory uncertainty. *Manage. Sci.* **63**, 497–518 (2016).
5. Unacast. Unacast Social Distancing Dataset. (2020). Available at: https://www.unacast.com/data-for-good.
6. Bürkner, P.-C. & Vuorre, M. Ordinal regression models in psychology: A tutorial. *Adv. Methods Pract. Psychol. Sci.* **2**, 77–101 (2019).
7. Bürkner, P.-C. Bayesian Item Response Modeling in R with brms and Stan. *arXiv Prepr. arXiv1905.09501* (2019).
8. Gelman, A. *et al. Bayesian data analysis*. (Chapman and Hall/CRC, 2013).
9. Niv, Y., Joel, D. & Dayan, P. A normative perspective on motivation. *Trends Cogn. Sci.* **10**, 375–381 (2006).
10. Behrens, T. E. J., Hunt, L. T., Woolrich, M. W. & Rushworth, M. F. S. Associative learning of social value. *Nature* **456**, 245–249 (2008).
11. Rutledge, R. B., Skandali, N., Dayan, P. & Dolan, R. J. A computational and neural model of momentary subjective well-being. *Proc. Natl. Acad. Sci.* **111**, 12252–12257 (2014).
12. Kang, M. J. *et al.* The Wick in the Candle of Learning. *Psychol. Sci.* **20**, 963–973 (2009).
13. Gureckis, T. M. & Markant, D. B. Self-Directed Learning: A Cognitive and Computational Perspective. *Perspect. Psychol. Sci.* **7**, 464–481 (2012).
14. Gruber, M. J., Gelman, B. D. & Ranganath, C. States of curiosity modulate hippocampus-dependent learning via the dopaminergic circuit. *Neuron* **84**, 486–496 (2014).
15. Wade, S. & Kidd, C. The role of prior knowledge and curiosity in learning. *Psychon. Bull. Rev.* **26**, 1377–1387 (2019).
16. Dubey, R. & Griffiths, T. L. Reconciling Novelty and Complexity Through a Rational Analysis of Curiosity. *Psychol. Rev.* **127**, 455–476 (2019).
17. Loewenstein, G. The psychology of curiosity: A review and reinterpretation. *Psychol. Bull.* **116**, 75 (1994).
18. MacKinnon, D. P., Krull, J. L. & Lockwood, C. M. Equivalence of the mediation, confounding and suppression effect. *Prev. Sci.* **1**, 173–181 (2000).
