# Supplementary material for: An energizing role for motivation in information-seeking during the early phase of the COVID-19 pandemic
Source: Nat Commun. 2022 Apr 28;13:2310. doi: 10.1038/s41467-022-30011-5 (PMC9050882; doi:10.1038/s41467-022-30011-5)
Supplement: Supplementary file 3 — Reporting Summary [file 41467_2022_30011_MOESM3_ESM.pdf]

nature research

Corresponding author(s): Yaniv Abir

Last updated by author(s): Mar 4, 2022

Reporting Summary

Nature Research wishes to improve the reproducibility of the work that we publish. This form provides structure for consistency and transparency in reporting. For further information on Nature Research policies, see our Editorial Policies and the Editorial Policy Checklist.

Statistics

For all statistical analyses, confirm that the following items are present in the figure legend, table legend, main text, or Methods section.

n/a	Confirmed	
☐	☒	The exact sample size (*n*) for each experimental group/condition, given as a discrete number and unit of measurement
☐	☒	A statement on whether measurements were taken from distinct samples or whether the same sample was measured repeatedly
☐	☒	The statistical test(s) used AND whether they are one- or two-sided<br>*Only common tests should be described solely by name; describe more complex techniques in the Methods section.*
☐	☒	A description of all covariates tested
☐	☒	A description of any assumptions or corrections, such as tests of normality and adjustment for multiple comparisons
☐	☒	A full description of the statistical parameters including central tendency (e.g. means) or other basic estimates (e.g. regression coefficient) AND variation (e.g. standard deviation) or associated estimates of uncertainty (e.g. confidence intervals)
☐	☒	For null hypothesis testing, the test statistic (e.g. F, t, r) with confidence intervals, effect sizes, degrees of freedom and P value noted<br>*Give P values as exact values whenever suitable.*
☐	☒	For Bayesian analysis, information on the choice of priors and Markov chain Monte Carlo settings
☐	☒	For hierarchical and complex designs, identification of the appropriate level for tests and full reporting of outcomes
☒	☐	Estimates of effect sizes (e.g. Cohen's d, Pearson's r), indicating how they were calculated

Our web collection on statistics for biologists contains articles on many of the points above.

Software and code

Policy information about availability of computer code

Data collection	Experiments were coded using jsPsych 6.1.0. The code used to run the experiments was deposited to OSF https://osf.io/gjcu9/
Data analysis	Analysis code was written in R 3.6.0, Stan 2.23.0 and Julia 1.4.2. The code used in our analyses and to reproduce the figures and the computational environment used to run the code were deposited to OSF https://osf.io/gjcu9/

For manuscripts utilizing custom algorithms or software that are central to the research but not yet described in published literature, software must be made available to editors and reviewers. We strongly encourage code deposition in a community repository (e.g. GitHub). See the Nature Research guidelines for submitting code & software for further information.

Data

Policy information about availability of data

All manuscripts must include a data availability statement. This statement should provide the following information, where applicable:
- Accession codes, unique identifiers, or web links for publicly available datasets
- A list of figures that have associated raw data
- A description of any restrictions on data availability

The entire dataset analyzed in this paper was deposited to OSF https://osf.io/gjcu9/. Source data for figures are provided with this paper.

Field-specific reporting

Behavioural & social sciences study design

All studies must disclose on these points even when the disclosure is negative.

Study description	Quantitative data. A cross-sectional sample of participants completed a waiting-for-answers task, rated questions on various dimensions, and completed self-report questionnaires. A week later they had to recall the answers they had waited for. Analyzed variables include binomial decisions and Likert scale ratings, all quantitative.
Research sample	US based participants recruited through Amazon Mechanical Turk. This is a convenience sample, representative of the varied population that uses MTurk, but not a sample representative of the US population. Median age 36, range 18-89; 2818 female. We chose to recruit on MTurk due to ease and speed of data collection, as well as the high variability of participants in terms of geographic spread, age, SES and other traits. This allowed us to measure across a wide spectrum of COVID-19-related motivational states.
Sampling strategy	Random sampling. Data was collected twice weekly, on Mondays and Thursdays. Throughout weeks 2-5 of data collection, 400 new participants were recruited on each day of data collection. In the last 3 weeks of collection this number was reduced to 300 per day for budgetary reasons. During the first week of data collection we were setting up data collection infrastructure, and so participant numbers were lower (652). Sample size on each day was not determined by statistical analysis. Instead, we opted for the maximal sample size our budget allowed.
Data collection	Participants completed the experiments at home, on their personal computers. Accordingly, no researcher was present at the time of data collection, nor was there an interaction between participants and a researcher at the time of data collection, and so blinding is non applicable.
Timing	Data was collected twice weekly, on Mondays and Thursdays, between March 11th and May 7th 2020.
Data exclusions	6135 participants completed session 1. Four participants reported technical difficulties in the presentation of questions. Their data was excluded from analysis. Data from 358 participants (5.84%) reporting less than perfect English language fluency, and 335 participants (5.46%) who interacted with other applications more than 5 times during the waiting or rating tasks were further excluded from analysis. Following previous studies with the waiting task, we excluded data from participants who failed to respond on more than 20% of trials (n=4, 0.07%), or whose mean response time was more than 2 standard deviations (SD) lower than the group average (n=58, 0.95%). Overall, data from 5376 participants was included in analyses.
Non-participation	We do not have data regarding participants who chose not to complete the experiment. 71.48% of eligible participants from session 1 also returned to complete session two.
Randomization	Participants were not allocated into groups. Order of blocks in the waiting task was determined randomly. Random allocation to groups is not applicable since participants were not divided into groups in this study.

Reporting for specific materials, systems and methods

We require information from authors about some types of materials, experimental systems and methods used in many studies. Here, indicate whether each material, system or method listed is relevant to your study. If you are not sure if a list item applies to your research, read the appropriate section before selecting a response.

Materials & experimental systems

n/a	Involved in the study	
☒	☐	Antibodies
☒	☐	Eukaryotic cell lines
☒	☐	Palaeontology and archaeology
☒	☐	Animals and other organisms
☐	☒	Human research participants
☒	☐	Clinical data
☒	☐	Dual use research of concern

Methods

n/a	Involved in the study	
☒	☐	ChIP-seq
☒	☐	Flow cytometry
☒	☐	MRI-based neuroimaging

Human research participants

Policy information about studies involving human research participants

Population characteristics	See above
Recruitment	Participants were recruited via Amazon Mechanical Turk, using the Cloud Research interface. Only participants resident in the United States, with a previous approval rating of at least 95% over at least 100 previous HITs were allowed to participate. An ad was placed on the Mechanical Turk platform as means of recruitment.<br>This sample is representative of the population that partakes in MTurk experiments, but not the general US population. Additional bias may have been introduced by participants dropping out if they find the task personally uninteresting. This may remove very low curiosity participants from our samples. Overall however, we see a wide range of responses on our tasks, including participants who indicated not to be interested in any of the questions presented as stimuli. Drop-out rates between the two session were relatively low, as reported in the main text.
Ethics oversight	Columbia University institutional review board.

Note that full information on the approval of the study protocol must also be provided in the manuscript.
